# European health literacy survey questionnaire short form (HLS-Q12): adaptation and evidence of validity for the Brazilian context

**DOI:** 10.1186/s41155-023-00263-1

**Published:** 2023-09-06

**Authors:** Daniela Sacramento Zanini, Evandro Morais Peixoto, Josemberg Moura de Andrade, Iorhana Almeida Fernandes, Maynara Priscila Pereira da Silva

**Affiliations:** 1 Pontifícia Universidade Católica de Goiás, Goiânia, Brazil; 2 Universidade São Fransisco, Campinas, Brazil; 3 Universidade de Brasília, Brasília, Brazil

**Keywords:** Health literacy, Public health, Validity evidence, Item response theory, Literacia em saúde, Saúde pública, Evidências de validade, Teoria de resposta ao item

## Abstract

Health literacy (HL) refers to knowledge, motivation and skills to understand, evaluate and apply health information, enabling appropriate decision making in daily life on health care and health promotion. Studies show that HL is associated with several social determinants, health outcomes, and health promotion. In Brazil, studies on the thematic are still scarce. Thus, the present study aimed to adapt, seek evidence of validity, reliability and estimate the parameters of the items of the European Health Literacy Survey Questionnaire Short Form (HLS-Q12) for the Brazilian context. 770 individuals participated, recruited through advertisements in the media and social networks, 82.1% female, aged between 18 and 83 (M = 35.5, SD = 13.52), from 21 Federative Units of Brazil and the Federal District. The subjects answered the HLS-Q12 and a sociodemographic questionnaire. Exploratory factor analysis indicated a unifactorial structure with good psychometric characteristics (GFI = 0.98; CFI = 0.98; RMSEA = 0.08; RMSR = 0.07). Cronbach's alpha, Guttman's lambda 2 and McDonald’s omega reliability indicators were equal to 0.87. We conclude that the HLS-Q12 is an adequate instrument to assess the level of HL in the Brazilian population.

The concept of Health Literacy (HL) emerged in 1974 in an article entitled ‘‘*Health education as social policy*’’, published by Simonds (1974). Initially, HL was related to performance in certain tasks, such as understanding health-related materials, prescriptions, medication labels, appointment scheduling cards, among others. In this sense, some studies treated the term as similar to lettering (Pedro et al., 2016; Silva & Jolluskin, 2017).

The report of the Ad Hoc* Council on Scientific Affairs* of the *American Medical Association* (1999) analyzed articles published between 1993 and 1998, with the participation of several health professionals. The report references HL as the ability to read and understand essential health-related materials necessary for patient functioning. The articles included in the report also pointed out that low literacy (in the sense of health literacy) promoted poor adherence to treatments and a worsening of cases, leading to hospitalization.

This definition has received some criticism arguing that HL is more than just reading and writing skills and, over the years, discussions related to the topic have suggested a broader perspective related to the ability to make informed decisions in daily life (Finbråten et al., 2018). Contributing to this process, in 1998 the World Health Organization (WHO) defined HL as the set of cognitive and social skills and the ability of individuals to understand and use information in order to promote and maintain good health.

In the year 2000, Nutbeam (2000) identified HL as a relatively new concept, defining it as a composite term to describe a series of outcomes in communication and health education activities. The author also distinguished three essential types of literacy, namely: basic or functional, interactive or communicative, and critical. Basic or functional literacy refers to the approach focused on the presence of reading and writing skills, allowing effective functioning in daily activities. This type of literacy is related to the first definitions of the term (Silva & Jolluskin, 2017). Literacy related to communication or interactive refers to cognitive skills associated with a set of social skills. Thus, this type can be used to actively participate in day-to-day activities, in order to gather information and deduce meaning from different forms of communication. Finally, critical literacy corresponds to the most advanced cognitive skills that, combined with social skills, can be applied for a critical analysis of information and to exercise greater control over life events and situations (Carvalho et al., 2016).

In order to contribute to the understanding of the thematic, Kickbusch et al. (2005) added social and life in society components to the definition of HL. Thus, the authors defined HL as the ability to make informed decisions in daily life, at home, in the community, in the workplace, in the use of health services, in the market and in the political context. Therefore, HL came to be understood, then, as a training strategy to increase people's control over their health, their capacity to seek information and to assume responsibilities.

Despite the different definitions of HL, it can be said that, commonly, the concept is related to knowledge, motivation and skills to access, understand, evaluate and apply health information to make judgments and decisions in daily life for care and health promotion (Sorensen et al., 2012). This definition and model cover the *continuum* in health that ranges from the well-being to the malaise of the individual in society. Within this perspective, recent studies have shown that HL is associated with several social determinants, health outcomes and health promotion (Azlan et al., 2021; Pavão & Werneck, 2021; Santos et al., 2017).

More specifically, individuals with low HL are less likely to understand information (written and oral) provided by health technicians, as well as less ability to navigate the health system to obtain the necessary services. Also, less likely to perform the necessary procedures and follow prescribed indications are identified (Arriaga et al., 2022; Pedro et al., 2016).

In a study carried out by Liu et al. (2020), the association between HL and the prevention of chronic diseases was investigated. For this purpose, a sample of 8,194 participants, aged between 15 and 69, from the 2017 National Health Literacy Surveillance Data from China was used. The authors suggested that HL is associated with a reduction in the likelihood of having a comorbid condition. However, this protective effect is only found among urban residents, suggesting that HL may be a key factor explaining the rural–urban difference. In this sense, we can hypothesize that HL has a moderator role in developing comorbid conditions when health services are available such as in urban spaces but not in rural, with a limited health service access.

Based on the importance of the construct and the need for evaluation, a consortium called *Consortium Health Literacy Project European* was created in Europe. This consortium developed an instrument for assessing HL called *Health Literacy Survey Questionnaire* (HLS-EU). This instrument aims to investigate, in an extensive and comparative way, HL in different European countries. From the information collected by this study, it was expected to develop more effective policies and interventions related to the health promotion of the population across Europe (Pelikan et al., 2019).

The HLS-EU is based on the conceptual model of Sorensen et al. (2012), in which three domains of health literacy are distinguished (health care, disease prevention and health promotion), and four categories of information management (accessing, understanding, appraising, and applying the information). These three domains and the four categories were combined (4 × 3) and resulted in twelve subdomains (Pelikan et al., 2019). The process of dividing the scale into domains, categories and subdomains was carried out from a theoretical perspective, that is, applying the conceptual model for the development of subdomains. However, the internal structure of the HLS-EU was tested and a three-factor structure (or domains) were confirmed (Sorensen et al., 2013). For each subdomain created, concrete tasks or behaviors were defined, so that respondents could assess whether it was “very easy” or “very difficult” to perform them. In total, the instrument had 47 items that must be answered on a four (4) point scale (HLS-EU Consortium, 2012; Sørensen et al., 2013).

Regarding the reliability of the HLS-EU in the language versions of the participating countries, Cronbach alphas ranging from 0.90 to 0.96 were observed (HLS-EU Consortium, 2012). Despite the good psychometric quality presented by the instrument, its high number of items, as well as its different subdomains, make its application difficult in population studies. Such aspects also make it difficult to adapt to different cultures.

Finbråten et al. (2018), when adapting the HLS-EU to the Norwegian context, observed some adjustment problems. Combining analysis of the psychometric properties of the items from the Rasch model and confirmatory factor analysis (CFA), the authors reduced the 47-item scale to a more parsimonious 12-item version. Items that had poor adjustments or were assessed as redundant were excluded. The final version of this instrument was named HLS-Q12.

For such HLS-Q12 adaptation study, Finbråten et al. (2018) randomly collected data from 900 individuals aged 16 and older. The HLS-Q12 showed acceptable fit for the unidimensional Rasch model and achieved acceptable goodness of fit indices using CFA. The H coefficient equal to 0.826 was indicated as a reliability index. According to the authors, this reduced version can contribute to a reliable measurement of behaviors related to HL, in addition to reducing the scale application time, which can be positive in large-scale research, due to the ease and speed of the individual in respond to the instrument. The instrument can also be used as a clinical screening tool for HL (Finbråten et al., 2018).

In Brazil, studies of instruments on HL are still scarce. Recently, in a study carried out by Mialhe et al. (2022), the objective was to analyze the psychometric properties of the Brazilian Portuguese version of the *European Health Literacy Survey Questionnaire*, 16-item version (HLS-EU-Q16). The study sample consisted of 783 Brazilian adults from three Federative Units (FU) (São Paulo, Fortaleza and Goiás) with a mean age of 38.6. In this study, the database was divided into two with random choice of participants. In this way, the databases were named as follows: complete sample (CS with 783 cases); sample 1 (S1—training bench with 392 cases) and sample 2 (S2—test bench with 391 cases). The model tested with cross validation indicated satisfactory, adequate, consistent, and stable levels for the instrument. The values for the reliability for the S1 were a Cronbach’s alpha and a McDonald’s Omega of 0.91; for S2, it was 0.92; and for the complete sample, it was 0.92 for both indicators. The authors concluded that the HLS-EU-Q16 is unidimensional, and adequate to measure HL in Brazilian adults.

The study by Mialhe et al. (2022) is an effective contribution to the measurement of HL, however, no adaptation studies of the HLS-Q12 (Finbråten et al., 2018) for Brazil were found. This shorter version of the scale is described in the literature as cost effective for measuring HL and provides the same one-dimension evaluation of HL as HLS-Q16. Therefore, in terms of a continental country like Brazil, with great socioeconomic and cultural differences, it is desirable to adapt instruments considering different regions of Brazil. Furthermore, the development of measures to assess literacy in the Brazilian population is necessary for good health planning and having shorter instruments will help assess a broad and diverse population in a shorter amount of time. Thus, this cross-sectional study, carried out from the third month of the Covid-19 pandemic in Brazil, aimed to adapt and seek evidence of validity based on the internal structure and the reliability estimates of the *European Health Literacy Survey Questionnaire Short Form* (HLS -Q12) for the Brazilian context. Although the study was conducted during the pandemic, no studies were found with the instrument that considered the effect of the period on the results. Therefore it is not possible to state any information about the implications of this period on HL. In addition, the parameters of the HLS-Q12 items were also estimated from the Item Response Theory (IRT).

The present study is justified considering that: (a) the theme of literacy has direct implications in the design of public policies and in different health outcomes; (b) an inadequate level of health literacy can have significant implications for the population's health indices, the use of health services and, consequently, investments in health; (c) the experience of the Covid-19 pandemic produced significant impacts on people's health (Zanini, Peixoto, Andrade, Campos et al., 2021a; Zanini, Peixoto, Andrade, & Tramonte, 2021b), many of them preventable through health education.

## Method

### Participants

770 individuals (82.1% women) participated in this study, aged between 18 and 83 (Mean = 35.5, SD = 13.52), from 21 Federative Units of Brazil and the Federal District. In terms of geographic macro-region, 18 (2.4%) respondents were from the North region, 241 (31.2%) from the Northeast region, 219 (28.4%) from the Midwest region, 222 (28.9%) from the Southeast region and 69 (9%) from the South region. As for the education level, 22% of respondents reported having completed high school, 24.7% incomplete higher education, 15.3% completed higher education, 10.8% incomplete postgraduate education and 46.1% completed postgraduate.

### Procedure

For the translation of the 12 items of the HLS-Q12, the methodological recommendations of Borsa et al. (2012) and of the *International Test Commission* (ITC, 2010) were followed. The items were translated from English to Portuguese independently by three psychologists who, in turn, discussed the translations and, by consensus, chose the 12 translated items. A fourth bilingual psychologist back-translated the items into English. Finally, the research group verified the semantic adequacy of the items, not identifying problems of an idiomatic nature.

With regard to the application of the questionnaires, the participants were recruited through advertisements in the media and social networks (*Facebook*, *Instagram*, *WhatsApp*, etc.). The invitation for voluntary participation should be attested by accepting the Free and Informed Consent Form, in which anonymity was guaranteed, the possibility of withdrawing at any stage of the research at no onus to the participant, as well as the contact with the researchers for more information about the study or if there were any complications arising from participation in the research. The participants responded to the instruments virtually (online), through the software *Google Forms*. This online questionnaire did not allow missing answers. All responses were recorded between May 4 and July 17, 2020, corresponding to approximately three months after the start of the pandemic in Brazil.

### Instruments

To achieve the objectives of this study, the following instruments were used:

*Health Literacy Scale—*12-item short version (*Health Literacy Survey Questionnaire-Short-Form 12*—HLS-Q12). The *scale* consists of 12 items that are scored on a Likert-type scale ranging from 1 “very easy” to 4 “very difficult”. The short version of 12 items was validated by Finbrånten et al. (2018), showing good reliability and adjustment indices to the unidimensional model, namely: χ^2^(df) 142.71 (54), *p* < 0.001; SRMR = 0.059; RMSEA (90% CI) = 0.089 (0.078 – 0.095); CFI = 0.951; Coefficient H = 0.826.

#### Sociodemographic and social isolation questionnaire

For this study, a questionnaire was developed to assess socio-demographic data, such as age, gender, marital status, family income level, etc. Questions were also developed regarding adherence to social isolation, motivation for adherence to social isolation, existence of people characterized as a risk group and children in the family group in social isolation, level of activity during social isolation, etc.

### Data analysis

Descriptive statistics were used in order to characterize the sample of respondents with the support of the statistical software *Statistical Package for the Social Sciences* (SPSS) 26.0. In order to obtain evidence of validity based on the internal structure, an exploratory factor analysis (EFA) was performed using the *Factor* software, version 12.03.02 (Ferrando & Lorenzo-Seva, 2017). To that end, the polychoric matrix and the Unweighted Least Squares (ULS) extraction method were considered. The implementation of this method is suggested when employing polychoric matrices for ordinal items (Asún et al., 2015), in addition to being more suitable for the distribution of non-normal data (Li, 2016). In the present study, the absence of multivariate normality was identified from the Mardia’s coefficient (1970), with the multivariate *Kurtosis* value equal to 201.09 (*p* < 0.001). The decision on the number of factors retained was based on Horn's parallel analysis technique (*Optimal Implementation*) (Timmerman & Lorenzo-Seva, 2011).

The model's adequacy of fit was evaluated using the following fit indices: *Root Mean Square Error of Approximation* (RMSEA), *Standardized Root Mean Square Residual* (SRMR), *Comparative Fit Index* (CFI) and *Goodness of Fit Index* (GFI). The adequacy of the indices was considered according to Brown's indications (2006): RMSEA < 0.08; SRMR < 0.08; CFI > 0.90 e GFI > 0.95. The stability of the factors was evaluated using the H index. H values above 0.80 indicate that the latent variable is well defined and is replicable in different samples. Still, in order to corroborate the suitability of the unifactorial model, the following indices were considered: *Item Unidimensional Congruence* (UniCo), *Explained Common Variance* (ECV) and *Mean of Item Residual Absolute Loadings* (MIREAL). The values of UniCo > 0.95, ECV > 0.85 and MIREAL < 0.30 were considered, which indicate that the data can be treated as essentially unidimensional (Ferrando & Lorenzo-Seva, 2018).

Factor Determinacy Index (FDI), Orion marginal reliability and Sensitivity ratio (SR) were used as indicators of the quality and effectiveness of factor score estimates. The FDI is a measure of correlation between the latent score and the factor score. Values above 0.80 indicate that factor scores are good proxies of the actual latent score. The Orion index indicates the reliability of the factor score of all participants on that factor (Damasio et al., 2021; Ferrando & Lorenzo-Silva, 2018). The SR can be interpreted as the number of different factor levels than can be differentiated on the basis of the factor score estimates. Values above 2 are expected for this index (Ferrando & Lorenzo-Silva, 2018).

To assess the reliability of the instrument, Cronbach's alpha (α) α, Guttman's Lambda 2 (λ) and McDonald’s omega (ω) indices (Hayes & Coutts, 2020) were used. For reliability estimates, SPSS 26.0 software was also used. Finally, to estimate the Samejima Gradual Response Model (SGRM), the RStudio software was used, through the “*mirt*” package (Chalmers, 2012).

## Results

The initial results indicated the suitability of the matrix for factoring, using the Kaiser–Meyer–Olkin criteria (KMO = 0.90; 95% CI: 0.87–0.91), and Bartlett's Sphericity Test [χ^2^ (66) = 4,706.1; *p* < 0.001], suggesting the suitability of the data to be used in the EFA. Kaiser's criterion indicated up to two factors, while Horn's parallel analysis indicated retention of one factor. Thus, a new EFA was performed by setting a factor. The results of this EFA, that is, the factor loadings, commonality and explained variance, plus the reliability indicators, can be seen in Table 1.

**Table 1:** Factor loadings, commonality, explained variance and accuracy indices of the Health Literacy Scale

**Item**	Factor loading	h^2^
1. Find information on treatment of illnesses you have?	0.69	0.48
2. Understand what to do in medical emergencies?	0.71	0.51
3. Judge the advantages and disadvantages of different treatment options?	0.67	0.45
4. Follow the instructions on medication?	0.73	0.53
5. Find information on how to manage mental health problems like stress and depression?	0.70	0.49
6. Understand why you need health screenings?	0.76	0.58
7. Judge if the information on health risks in the media is reliable?	0.60	0.36
8. Decide how you can protect yourself from illnesses based on advice from family and friends?	0.40	0.16
9. Find information on health attitudes such as exercise, healthy food and nutrition?	0.76	0.57
10. Understand information on food packaging?	0.62	0.38
11. Judge which everyday behaviors are related to health?	0.78	0.61
12. Make decisions to improve your health?	0.59	0.34
Explained variance (%)	49.98%	
Cronbach's alpha (α)	0.87	
Guttman's Lambda 2 (λ)	0.87	
McDonald’s omega (ω)	0.87	

h^2^: commonality

The unidimensional model proved to be adequate, since the adjustment indices were considered good (GFI = 0.98; CFI = 0.98; RMSEA = 0.08; RMSR = 0.07), with factor loadings within the expected range, ranging from 0.40 (item 8—Decide how you can protect yourself from illnesses based on advice from family and friends?) and 0.79 (item 11—Judge which everyday behaviors are related to health?). The UniCo index equal to 0.98, ECV equal to 0.88 and MIREAL equal to 0.21 confirmed the unifactorial structure of the instrument (Ferrando & Lorenzo-Seva, 2018). It is also noteworthy that the model was able to explain 49.97% of the total data variance. The H-Observed index was equal to 0.87 indicating stability of the factor structure. Regarding the quality and effectiveness indicators of the factorial score estimates, good indices were observed: FDI = 0.96; Orion marginal reliability = 0.92; SR = 3.34.

The reliability indicators showed satisfactory values of 0.87, also for Cronbach's alpha, Guttman's Lambda 2 and McDonald's omega. Such results support the first evidence of validity based on the internal structure and the reliability estimate of the internal consistency of the Brazilian version of the HLS-Q12*.*

Considering the unidimensionality of the instrument, the psychometric properties of the items of discrimination (parameter *a*) and difficulty in endorsing or agreeing (parameter b) were estimated from the IRT. The results are shown in Table 2.

**Table 2:** Discrimination parameters and item difficulty

**Item**	*a*	*b*_*1*_	*b*_*2*_	*b*_*3*_
Item 01	1.72	-0.12	1.41	2.88
Item 02	1.81	-0.85	0.86	2.22
Item 03	1.63	-1.45	0.43	2.13
Item 04	2.01	0.38	1.67	2.62
Item 05	1.75	-0.32	0.97	2.31
Item 06	2.26	0.16	1.40	2.46
Item 07	1.31	-1.24	0.58	2.14
Item 08	0.76	-1.91	0.57	3.01
Item 09	2.12	0.18	1.53	2.85
Item 10	1.35	-0.31	1.08	2.32
Item 11	2.32	0.04	1.39	2.48
Item 12	1.27	-0.73	1.01	2.53

*a*: item discrimination parameter, *b*_*1*_ = threshold difficulty level for response categories 1 and 2; *b*_*2*_ = threshold difficulty level for response categories 2 and 3; *b*_*3*_ = threshold difficulty level for response categories 3 and 4

**Fig. 1:**
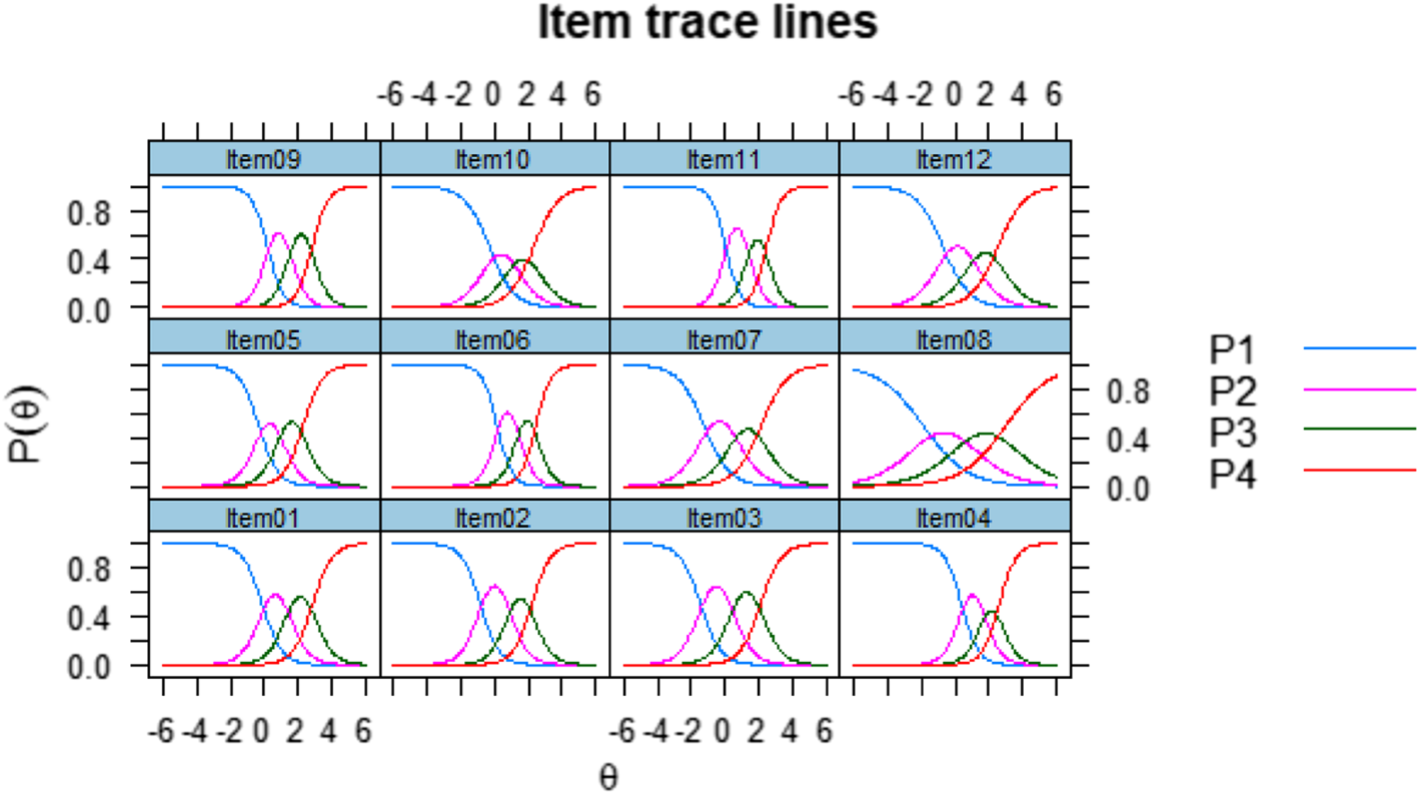
Item characteristic curves

From the results presented in Table 2, we can interpret that the discrimination (parameter *a*) of the items ranged from 0.76 (item 8) to 2.32 (item 11). This last item (item 11 = “Judge which everyday behaviors are related to health?”) is the item that most differentiates individuals with levels close to the latent trait (literacy). We also verified that the items covered a wide variation of the latent trait with thresholds (*b*) between -1.91 (*b*1 of item 8) and 3.01 (*b*3 of item 8). Next, in Fig. 1, the Characteristic Curves of the Items are presented.

**Fig. 2:**
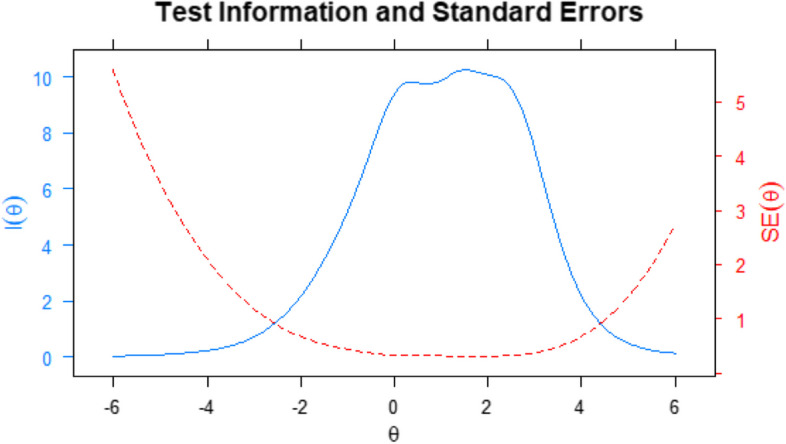
Information curve

From Fig. 1, we have a view of the 12 characteristic curves of each item on the Literacy scale. We can interpret that, for most items, each of the four possible response categories at some point on the θ scale had a higher probability of endorsement than the others. Next, in Fig. 2, the Test Information Curve is presented.

From the total information curve, we can observe that the instrument has more psychometric information than the error between the theta (θ) of -2.5 and + 4.0, approximately. That is, it is in this range of θ or scores that the HLS-Q12 best evaluates the Literacy construct.

## Discussion

The present study aimed to cross-culturally adapt the Health Literacy Survey (HLS-Q12), as well as to estimate its psychometric properties for the Brazilian context. In general, the results demonstrated the adequacy of the instrument, providing support for measuring the level of HL in the Brazilian population through a unifactorial structure, with good levels of reliability.

The results of the present research are similar to the study by Finbråten et al. (2018). Although the authors indicated the three-factor structure as more adequate compared to the one-factor structure, the unifactorial model also presented satisfactory fit indices (CFI = 0.95; NNFI = 0.94; RMSEA = 0.08 [0.07–0.09]). In addition, the accuracy indicators observed in the present study (α, λ, ω = 0.87) are consistent with those observed in the original study (0.82) (Finbråten et al., 2018), as well as in the Portuguese version (0.96) (Pedro et al., 2016), demonstrating the ability of the scale to measure the level of literacy with a low level of error, associated with the estimation of the target construct (Finbråten et al., 2018; Pedro et al., 2016; Tabachnick & Fidell, 2019).

The findings are similar to studies conducted for the French version (Roquette et al., 2018) and Brazilian version (Mialhe et al., 2022). In the study of the French version, the results suggested the unidimensionality of the instrument through Confirmatory Factor Analysis, as it presented adequate fit indices, suggesting that the model fit the sample data (CFI = 0.948; TLI = 0.913, and RMSEA = 0.176), with satisfactory internal consistency (α = 0.81). In the Brazilian version, Confirmatory Factor Analysis, Exploratory Factor Analysis, and IRT were employed, which support the unidimensionality. It is understood that the data from previous studies and the present study support the perspective that the instrument is capable of measuring health literacy through a single factor.

Furthermore, a study was conducted by Mialhe et al. (2021) for a shorter version of the instrument, consisting of six items, applied in two different samples. For both samples, the data suggested unidimensionality, with the first presenting factor loadings ranging from 0.67 to 0.80, with an explained variance of 69.92% of the latent variable. The second sample presented loadings between 0.64 and 0.83 and an explained variance of 71.23%. Confirmatory Factor Analysis was also applied, which added validity evidence to the one-factor structure, indicating that the models for both samples fit well with the data: sample 1 (CFI = 0.98; GFI = 0.99; RMSEA = 0.08) and sample 2 (CFI = 0.98; GFI = 0.98; RMSEA = 0.08).

From the IRT analysis, it was possible to identify that the items of the Literacy Scale are able to discriminate subjects with similar magnitudes of the latent trait, since the discrimination parameters (*a*) presented values above 0.60. It is understood that values between 0.60 and 1.8 are considered adequate for the composition of a good instrument, the higher the value of *a* the better the item will be considered (Andrade et al., 2010, 2021; de Ayala, 2022; Nakano et al., 2015). Similar to the results found, the research by Finbråten et al. (2018), also suggested the adequacy of the items to compose the set for measuring the construct, with the exception of two items that were more difficult to endorse.

The HLS-Q12 can be considered a necessary measure, as it can assist healthcare professionals in understanding the difficulty subjects have in comprehending, evaluating, and applying health information in different circumstances, such as the pandemic context. However, based on the findings and studies conducted during the pandemic period (e.g., Mialhe et al., 2021, 2022), it is not possible to state any information about the implications of this period on health literacy assessment. This is due to a lack of data on the relationship between the construct and the period, meaning there is a limitation to identifying the impacts of the pandemic on the results.

Having instruments that effectively assess HL can help the health system to be more proactive, providing directions for intervention strategies that can meet the needs of individuals who use this service (Sudore et al., 2009). This research, by proposing an instrument capable of measuring HL in Brazil, may be able to identify its levels. For example, low levels of literacy are associated with difficulty in developing and taking responsibility for their own health, including also participating in decision-making within this context (Finbråten et al., 2018).

## Conclusion

Therefore, the present research contributes to health professionals, by bringing a reflection on the construct. These professionals can map the literacy of their patients, identifying the best strategy to increase their levels. The present study had a wide and diverse sample of individuals from all regions of Brazil. Despite the results are significant and relevant for Brazil, there are some limitations that need to be remedied, such as the application of the instrument in longitudinal studies and with applications of interventions to understand the effect of the construct on the participants. It should also be noted that the present research sought to estimate evidence of validity based on the internal structure, and its relationships with other variables were not verified (AERA et al. 2014; Andrade & Valentini, 2018). Thus, it is recommended to analyze HL with other variables associated with health.

We emphasize that the application of the questionnaire took place exclusively online and further studies with face-to-face applications are needed. Equivalence studies with both types of applications are required (Schoot et al., 2012). In the present study, good indicators of quality and effectiveness of the factor score estimates were observed.

It is concluded that the HLS-Q12 is an adequate instrument to measure the level of HL in the Brazilian population with psychometric properties comparable to the Portuguese version. As indicated by Arriaga et al. (2022), HL has become an essential concept in public health. The authors point out that in Europe there is a high variability of HL levels in the countries. We believe that this reality also applies to Brazil, a country of continental dimensions.

## Data Availability

The datasets used and/or analysed during the current study are available from the corresponding author on reasonable request.

## Abbreviations

CFA: Confirmatory Factor Analysis
CFI: Comparative Fit Index
ECV: Explained Common Variance
EFA: Exploratory factor analysis
FDI: Factor Determinacy Index
GFI: Goodness of Fit Index
HL: Health Literacy
HLS-EU: Health Literacy Survey Questionnaire
HLS -Q12: European Health Literacy Survey Questionnaire Short Form
HLS-EU-Q16: European Health Literacy Survey Questionnaire, 16-item version
IRT: Item Response Theory
KMO: Kaiser–Meyer–Olkin
MIREAL: Mean of Item Residual Absolute Loadings
NNFI: Nonnormed Fit Index
RMSEA: Root Mean Square Error of Approximation
SGRM: Samejima Gradual Response Model
SPSS: Statistical Package for the Social Sciences
SRMR: Standardized Root Mean Square Residual
UniCo: Item Unidimensional Congruence
SR: Sensitivity ratio
ULS: Unweighted Least Squares
