# Correction: European health literacy survey questionnaire short form (HLS-Q12): adaptation and evidence of validity for the Brazilian context

**DOI:** 10.1186/s41155-023-00278-8

**Published:** 2023-11-08

**Authors:** Daniela Sacramento Zanini, Evandro Morais Peixoto, Josemberg Moura de Andrade, Iorhana Almeida Fernandes, Maynara Priscila Pereira da Silva

**Affiliations:** 1 Pontifícia Universidade Católica de Goiás, Goiânia, Brazil; 2 Universidade São Fransisco, Campinas, Brazil; 3 Universidade de Brasília, Brasília, Brazil

**Correction: Psicologia: Refexão e Crítica 36, 25 (2023)**

**https://doi.org/10.1186/s41155-023-00263-1**

Following publication of the original article (Zanini et al., 2023), the editor reported that the non-English abstract was not met the requirement in the journal submission guideline, which has been deleted.

The original article (Zanini et al., 2023) has been updated.
